# Five-year experience of African Vaccination Week implemented by the WHO Regional Office

**Published:** 2018-08-02

**Authors:** Joseph Okeibunor, Richard Mihigo, Blanche Anya, Felicitas Zawaira

**Affiliations:** WHO Regional Office for Africa, Brazzaville, Congo

**Keywords:** Africa, Vaccination week, Immunization, Vaccines

## Abstract

The 5^th^ edition of the African Vaccination Week (AVW) kicked off in Lusaka, Zambia, on 23 April 2016, the same day as did the 4th World Immunization Week (WIW), and vaccination week in other WHO regions. The theme was “Save lives, prevent disabilities, vaccinate!”. The aim was to draw attention to the need to attain universal immunization coverage in the African Region by closing the immunization gap, while also celebrating the important polio eradication milestone reached in the African Region.

Twenty-eight (59.6%) of the 47 countries in the African Region celebrated the AVW within the regionally set dates of 24^th^ to 30^th^ April 2015. However, given its flexibility, the celebration continued until September in 15 other countries in the Region. Three countries, namely Comoros, Gabon, and Cape Verde did not join the celebration for the 2015 edition of the AVW.

Countries used the opportunity to introduce new vaccines into their routine immunization. Populations, hitherto unreached with basic health services were reached with needed services, such as vitamin A supplementation, deworming, and catch up immunization services. The programmes promoted awareness of the benefits of vaccines and the rights of communities to demand vaccines and immunization services to save lives and prevent disabilities. The number of participating countries rose steadily from 40 in 2011 to 43 and 46 countries in 2013 and 2014 respectively. The number ranged from one intervention integrated with AVW in 17 countries to 5 interventions integrated with the AVW in three countries. In 2015, 67.4% of the participating countries integrated other interventions with AVW activities.

## Introduction

Vaccines have been recognized as one of the successful public health interventions to protect lives^1^. Tremendous progress has been made in the development of new vaccines, along with increasing access to existing globally. In the African Region vaccines have contributed tremendously to the eradication of diseases, the most recent being the interruption of wild poliovirus transmission^1^–^9^. Given the positive impact of childhood vaccination in preventing disease, disability, and death associated with polio^10^, perceived risks from vaccine-preventable diseases are presumed to have diminished significantly as parents recognized the effectiveness of vaccines^11^. The success story of vaccination against vaccine-preventable diseases has elicited demand that all children should have equal access to effective childhood vaccines to give every child a chance in life^10^,^12^.

In the African Region, like other WHO Regions in the world, childhood vaccine services are delivered primarily through national routine immunization programmes, using national immunization schedules administered at established locations and in fairly regular periods^13^,^14^. However, given a plethora of logistic, geographical and cultural challenges the routine programmes are unable to realize vaccination or target disease targets supplemental immunization activities (SIAs) such as campaigns have been conducted. For instance, the polio eradication initiative relied heavily on SIAs, which are usually are intensive, short duration activities designed to provide supplemental doses of a particular vaccine rapidly^13^. Another strategy that uses the campaign mood for providing all the recommended childhood immunizations, which has also become common in recent times, is the Periodic Intensification of Routine Immunization (PIRI)^14^,^15^.

PIRI share some characteristics of campaigns, but beyond accelerating disease control activities, it has the goal of raising routine vaccination coverage in a given geo-cultural entity. PIRI include activities such as Child Health Days and Immunization Weeks, used increasingly to supplement routine vaccination services, raise coverage, and reduce the number of missed and unvaccinated children as well as the number of children whose vaccination status is incomplete. In 2009, 109 countries reported using at least one supplemental vaccination delivery strategy^14^. This strategy has been formalized by the global and regional committees involving member states and utilized in furthering the course immunization programmes between 2010 and 2015.

One of the principal challenges of immunization programmes in the African Region has been to deliver effective vaccines to all populations, irrespective of social class and location and effectively contain vaccine preventable diseases with existing vaccines. In the last 5 years, the World Health Organization’s Regional Office for Africa (WHO-AFRO) has taken more practical steps to reach the unreached by applying core principles of primary health care, by enhancing coverage through advocacy, community mobilization, and participation. This strategy employed in engaging the people is a form of PIRI called African Vaccination Week (AVW). The AVW provides a platform for the Member States to speak in one voice, advocating for immunization as a public health priority in the Region, and deliver immunization services to larger populations. The initiative targets people with limited access to regular health services, thereby working to close the gaps in immunization^16^. The 60^th^ session of the WHO Regional Committee for Africa endorsed this initiative with a resolution (AFR/RC60/14), institutionalizing annual AVW for sustained advocacy, expanding community participation and improving immunization service delivery. The World Health Assembly, in its resolutions WHA58.15 and WHA61.15, also gave a global endorsement of the World Immunization Week.

The primary objective of African Vaccination Week (AVW) is to increase public awareness on the benefits of vaccines. Activities of the AVW programme address complementary aims of awareness and priority of vaccination in communities and among health professionals. Reducing demand-side constraints and promoting vaccination priority among healthcare providers, that is reducing missed opportunities for vaccination, together contribute to improved vaccination status of communities. By engaging health system and government policymakers, this is also intended to help strengthen priority and availability of resources for materials (availability of vaccines) and well-functioning services.

In addition to this focus on improved vaccination coverage, however, the AVW serves as a platform for the provision of other public health services to otherwise unreached segments of the population in the African Region. Since it was first launched in 2011, AVW has grown to become not only the largest multi-country immunization effort in the Region, with the participation of virtually every country in the Region, but also an important means of strengthening a wide range of public health activities^16^. The objective of this paper is to documents country experiences and lessons learned from implementing AVW in the 2010 to 2015. Such lessons are germane to ensuring more efficient use of the AVW in furthering the goals of the Global Vaccine Action Plan (GVAP), the Regional Vaccine Action Plan (RVAP) and optimizing the synergy derived from integration of public health interventions in the AVW platform^16^.

## African Vaccination Week

Though a regional event, the actual implementation of AVW is designed to allow countries some flexibility in defining their objectives, targets, and approaches, focusing on goals of national health strategies. The planning for each of the five past editions of AVW comes from the countries with inputs from immunization partners, under the coordination of the Regional Office. The countries develop their plans, enumerating the different activities to be undertaken during the week with targets. The plans are shared with the Regional Office. Teleconferences are held to discuss the plan and one planning workshop is held in the WHO/AFRO, Brazzaville to finalize the plans.

The AVW aims at increasing understanding of the value of vaccines and creates demand for immunization both as a right and responsibility of individuals and communities within the member states in line with the Global Vaccine Action Plan (GVAP)^17^. It creates awareness on the right of every person to be adequately protected against vaccine-preventable diseases. The implementation follows definite guidelines. A list of frequently asked questions as well as reporting templates are also developed to guide the implementers^16^. Activities planned in each country also reinforce the priority of measuring outcomes.

Often, countries seize the opportunity provided by the AVW to undertake an integrated delivery of public health interventions. Typically, the activities undertaken during the week include immunization (periodic intensified routine immunization and in catch-up modes to reach those missed with immunization services during the general campaign period). Other activities that get added on to the creation of awareness on the values of vaccines include vitamin A supplementation; water, sanitation and hygiene (WASH); deworming; distribution of long lasting insecticide treated bednets; testing for human immunodeficiency virus (HIV) and malaria. These activities target all (i.e., infants, children, adolescents, adults, women, and the elderly) depending on country priorities.

## Implementation of AVW since inception

### Country participation

In 2011, 40 of the 46 countries in the Region, then, organized various activities, such as vaccination of all ages against vaccine-preventable diseases and provision of various life-saving interventions, particularly for vulnerable groups and remote communities that traditionally lack access to health services. AVW reports over the first 3 years (2011 to 2013) show that >180 million people were vaccinated with OPV in 19 countries and the third year, 2013 edition, approximately 7.5 million doses of various vaccines were administered in 17 countries. Also, 31.5 million vitamin A and 21.2 million deworming tablets were distributed in 13 and nine countries respectively; a total of 6.4 million children were screened for malnutrition in Cameroon, Madagascar, Mozambique; and 3.8 million WASH kits were distributed in Botswana, Cameroon, and Nigeria.

The 2013 and 2014 AVW, the third and fourth editions respectively showed great improvement over the first and second editions marked in 2011 and 2012 respectively. The improvements were recorded in a number of countries involved and the delivery of services. In 2013, all countries except Central Africa Republic (CAR), Equatorial Guinea and Namibia participated in the AVW with the theme “Save lives, prevent disabilities, vaccinate!” between 22 and 28 April 2013. Participating countries also embarked on mobilization and sensitization campaigns, using traditional, modern and social media, engaging religious leaders, organizing sensitization workshops for media practitioners and health workers, among others. They also conducted community dialogues through panel discussions and recognized deserving health workers with award of certificates. They sensitized supervisors and undertook supportive supervisory visits to vaccination sites. These activities had a common central goal of projecting the prominent role and benefits of vaccination in promoting public health^16^.

The fifth edition of the AVW was launched in Lusaka, Zambia on 23 April 2015 and was observed between 24^th^ and 30^th^ April 2015. Twenty-eight (59.6%) of the 47 countries in the African Region celebrated the AVW within the regionally set dates of 24^th^ to 30^th^ April 2015. However, given its flexibility, the celebration continued until September in 15 other countries in the Region. Three countries, namely Comoros, Gabon, and Cape Verde did not join the celebration for the 2015 edition of the AVW.

As in previous years, reports from the Member States indicate that countries used the occasion to hold round-table discussions, advocacy, and social mobilization activities for immunization. They also held training sessions on various immunization activities such as ‘catch-up’ vaccination or campaigns against vaccine-preventable diseases, the introduction of new vaccines into national routine immunization programmes. Furthermore, the period was used for the provision of life-saving interventions such as deworming, vitamin A supplementation, distribution of mosquito nets, growth monitoring, HIV and Malaria testing (Table 1).

Countries participating in each edition employed various traditional and social media to sensitize, mobilize and engage religious leaders, media practitioners, and health workers, among others in promoting the values of vaccines and immunization demand. The countries conducted community dialogues through panel discussions and recognized deserving health workers through the award of certificates and sensitize supervisors as well as undertook supportive supervisory visits to vaccination sites. These activities had a common goal: to show the benefits of vaccination in promoting public health.

In addition to this, Mihigo et al.^16^ reported that the countries used the AVW to introduce some new vaccines like the pneumococcal conjugate vaccine, rotavirus, and human papillomavirus (HPV) vaccines and provision of other health commodities as part of ‘catch up’ activities in low-performing districts. They also noted that countries conducted polio and measles campaigns, screened children for moderate or severe malnutrition, distributed water sanitation and hygiene (WASH) kits, oral rehydration salt (ORS), iron and folate tablets as well as condoms. These activities served several purposes. Some of the, include raising awareness on the life-saving value of immunization; reaching underserved and marginalized communities (particularly those living in remote areas, deprived urban settings and strife-torn areas) with high-impact child survival packages. Others include reinforcing the medium and long-term benefits of immunization and other child survival interventions, all with the aim of increasing vaccination coverage and helping to transform the lives of millions of children, by giving them a chance to grow up healthy, go to school, and improve their life prospects.

A wide range of vaccination activities was implemented under the umbrella of AVW. These include intensification of the routine programme to complete vaccination schedules: polio eradication campaigns, catch-up vaccination activities (Table 1). More than half (55.8%) of the 43 participating countries integrated other interventions into the AVW activities for 2013. Interventions integrated, however, varied in number and type. The number ranged from one in Algeria, DRC, Kenya, Lesotho, Mali, Mauritania, Rwanda and Togo, to five in Cameroon. In-between, Angola, Burkina Faso, Chad, Congo, Nigeria, Swaziland and Zimbabwe integrated two interventions, while Benin, Côte d’Ivoire, Gabon and Liberia integrated three interventions. Another set of countries integrated four interventions. These included Guinea, Madagascar, and Mozambique. In 2014 all the participating countries integrated one intervention or another with the AVW activities. The number ranged from one intervention integrated with AVW in 17 countries to 5 interventions integrated with the AVW in three countries. In 2015, 67.4% of the participating countries integrated other interventions with AVW activities. In one Eritrea as many as six interventions were integrated with the AVW 2015 edition. Others integrated between one and five interventions.

Table 2 presented the countries on the conduct of non-immunization related interventions such sensitization, health talks, social mobilization and other health communication activities. Other interventions include Vitamin A administration, deworming and nutritional interventions. Countries like Madagascar implemented all the non-immunization related activities during the AVW.

The most common interventions integrated with AVW activities in 2015 included immunization catch-up, integrated vitamin A supplementation and deworming. These activities were integrated with AVW activities in 19 countries each. Child health days were also conducted in eight countries and distribution of long-lasting insecticide-treated bednets in four countries (Table 2). Polio eradication activities were integrated into three countries while integrated management of childhood illness (IMCI) was integrated into two countries. Kenya took advantage of the celebration to launch it’s National Immunization Technical Advisory Group (NITAG).

## Discussion

Since the inception of AVW, increasingly more countries in the Region have participated in its celebration. Countries have used the AVW as a platform for reaching the people, previously unreached, with key public health interventions. They have also used the opportunity of the AVW to create awareness on immunization and conducted advocacy to government and other stakeholders.

For the first 2 years of AVW, that is 2011 and 2012; there were hardly any attempts to document the activities. The focus of the key players was to increase coverage using what they considered a veritable platform for an integrated and comprehensive public health service targeting hard to reach groups along with the introduction of new vaccines to contribute towards efforts to attain the health MDGs^16^. Consequently, we have not data to present in those 2 years. As the initiative recorded successes, albeit undocumented, subsequent editions were then planned to ensure proper documentation both for purposes of evaluation and dissemination of best practices. Since then conscious efforts have been made to document country experiences with the initiative each of the last three years 2013 to 2015.

Lessons learnt from the 2013 and 2014 AVW indicate that it is a viable strategy for reaching programme goals. There was an increase in the number of countries from participating in the initiative, from 40 to 47 in 2013 and 2014 respectively. Similarly, interventions implemented with AVW increased from 7 in 2013 to 19 in 2014. It shows the appreciation of the feasibility of driving the integration goals on the AVWs. The preceding is in itself is a success story for the AVW16. However, the results in 2015 showed a downward trend from 19 in 2014 to 17 in 2015, which has been attributed to a number of factors.

A major reason for the drop is blamed of the consequence of the Ebola virus disease (EVD) outbreak, which threatened the health system in Africa. Many health care resources in the Region were channeled to interrupt EVD transmission in the three most affected countries. The other countries in the West African sub-Region, though not part of the most affected, also suffered some effects of the EVD outbreak in Guinea, Liberia, and Sierra Leone. We also experienced data problems in the 2015 exercise, which is also a fall out of the Ebola epidemic and data managers were pulled to focus on the Ebola outbreak and other public health emergencies in the Region. Fragility of the health systems in response to Ebola and other major events affecting capacity of health systems is acknowledged. Limited capacity and reach of primary health services are among the factors motivating AVW platform. These constraints, notwithstanding, the platform is still very effective in ensuring that public health services and commodities reach the previously unreached in an integrated manner.

This paper is based on reports and programme records. Though verifiable and verified for validity and reliability, the paper lacks the benefits of a deliberately designed survey to interact with recipients. The manipulation of data was very minimal. There will be a need to ensure a multidisciplinary team to achieve in-depth analysis. AVW is set against universal public standards, which is lacking in most African settings, where many health systems are weak^18^,^19^. There is also the cultural limitation of record keeping. The programmes had a weak culture of record keeping and documentation. The Regional Routine Immunization Officer, however, managed to follow up to gather enough data to at least describe the situation. It is planned that in the future a more elaborate record keeping systems to conduct evaluations.

In conclusion, the AVW has been proven to be a useful platform for providing key public health interventions. More important is that it has also enhanced integrated delivery of public health interventions, thereby optimizing the use of scarce resources available for the delivery of services in the Region. It is thus recommended that AVW should be promoted and used as a platform for ensuring integration in service delivery.

## Figures and Tables

**Table 1:** Main immunization related activities Conducted in 2013, 2014 and 2015 AVWs by Countries.

Country	Polio Campaign			Catch up vaccination			Vitamin A administration			New Vaccine Introduction				Total
										Rotavirus	Pneumococcal conjugate vaccine	Human papillomavirus vaccine	Measles 2nd dose	
	2013	2014	2015	2013	2014	2015	2013	2014	2015	2013	2014	2014	2014	
Algeria				X	X	X			X					4
Angola				X	X	X	X	X	X	X				7
Benin	X	X		X	X	X	X		X					7
Botswana					X			X						2
Burkina Faso	X	X		X	X	X			X					6
Burundi						X			X					2
Cabo Verde					X									1
Cameroon	X			X			X	X						4
Central African Republic		X			X	X			X					4
Chad	X			X	X	X			X					5
Comoros					X			X						2
Congo				X	X	X	X		X	X				6
Cote D'Ivoire	X				X	X	X		X					5
DRC				X	X	X			X					4
Equatorial Guinea		X												1
Eritrea			X		X	X		X	X					5
Ethiopia					X	X			X					3
Gabon				X	X		X							3
Ghana					X			X						3
Guinea	X		X	X	X	X	X	X	X					8
Guinea Bissau					X	X			X					3
Kenya				X										1
Lesotho				X	X						X			3
Liberia	X		X			X	X	X	X					6
Madagascar				X	X		X	X						4
Malawi					X									1
Mali	X	X				X			X					4
Mauritania				X		X		X	X					4
Mauritius														0
Mozambique	X				X		X	X						4
Namibia					X			X						3
Niger		X												1
Nigeria					X		X	X						3
Rwanda							X	X				X		3
Sao Tome & Principe					X			X						2
Senegal					X	X			X					3
Seychelles												X		1
Sierra Leone	X													1
South Africa														0
South Sudan								X						1
Swaziland				X	X		X				X			4
Tanzania												X	X	2
The Gambia		X						X						2
Togo				X	X			X						3
Uganda					X	X			X					3
Zambia					X			X						2
Zimbabwe				X	X									2
**TOTAL**	**10**	**7**	**3**	**17**	**32**	**19**	**13**	**20**	**19**	**2**	**2**	**3**	**1**	**148**

**Table 2:** Non-Immunization related activities undertaken during AVW in 2013, 2014 and 2015.

Couintries	Advocacy & sensitzation		Short messages services	Deworm children			Malnutrition screening			Child health days		Bednet distribution		Total
	2014	2015	2014	2013	2014	2015	2013	2014	2015	2014	2015	2014	2015	
Algeria	X	X				X								3
Angola	X	X			X	X						X		5
Benin	X	X				X								3
Botswana	X	X			X					X	X			5
Burkina Faso	X					X								2
Burundi	X	X				X					X			4
Cabo Verde	X													1
Cameroon	X	X		X	X		X			X	X			7
CAR	X	X				X								3
Chad	X	X				X		X				X		5
Comoros	X				X									2
Congo	X	X				X						X		4
Cote D'Ivoire	X	X		X		X			X				X	6
DRC	X	X				X								3
Equatorial Guinea	X	X												2
Eritrea	X	X				X			X		X			5
Ethiopia	X	X				X								3
Gabon	X			X										2
Ghana	X	X						X		X	X			5
Guinea	X	X		X	X	X								5
Guinea Bissau	X	X				X						X		4
Kenya	X	X	X										X	4
Lesotho	X	X												2
Liberia	X	X		X	X	X								5
Madagascar	X	X		X	X		X	X		X	X			8
Malawi	X	X												2
Mali	X	X				X								3
Mauritania	X	X	X		X	X		X						6
Mauritius	X	X												2
Mozambique	X	X		X	X		X							5
Namibia	X	X												2
Niger	X	X												2
Nigeria	X	X		X	X					X	X			6
Rwanda	X	X			X			X		X	X			6
Sao Tome & Principe	X	X												2
Senegal	X	X				X								4
Seychelles	X	X												2
Sierra Leone	X	X									X			3
South Africa	X	X												2
South Sudan	X	X												2
Swaziland	X	X			X								X	4
Tanzania	X	X												2
The Gambia	X	X			X									3
Togo	X	X			X									3
Uganda	X	X	X			X								4
Zambia	X	X			X								X	4
Zimbabwe	X	X		X										3
**TOTAL**	**47**	**43**	**3**	**9**	**15**	**19**	**3**	**5**	**2**	**6**	**11**	**4**	**4**	**170**
